# Osteoid Osteoma on the Distal Phalanx of the Ring Finger: A Rare Case Report

**DOI:** 10.7759/cureus.97600

**Published:** 2025-11-23

**Authors:** Motoi Hayashi, Kazuhiko Hashimoto, Shunji Nishimura, Kazuhiro Ohtani, Koji Goto

**Affiliations:** 1 Orthopedic Surgery, Kindai University Hospital, Osakasayama, JPN

**Keywords:** diagnosis, elderly, osteoid osteoma, ring finger, surgical treatment

## Abstract

Osteoid osteoma (OO) is a benign osteoblastic lesion and a relatively common bone tumor; however, its diagnosis can be challenging depending on the site of occurrence. We present a rare case of OO located in the distal phalanx of the right ring finger in a 23-year-old male. The patient experienced pain and swelling beginning two months after sustaining a finger contusion. Radiographic evaluation, including X-rays and CT, revealed a radiolucent lesion with central calcification characteristic of OO. Surgical excision was performed, preserving the nail bed, and histopathological analysis confirmed the diagnosis. At the two-month follow-up, there was no evidence of recurrence. This case underscores the importance of including OO in the differential diagnosis of fingertip bone lesions. Complete surgical resection can lead to excellent clinical outcomes without functional compromise.

## Introduction

Osteoid osteoma (OO) is a benign bone-forming tumor characterized by its small size and limited growth potential [1]. Approximately 50% of OO cases affect long bones, particularly the femur and tibia [1]. Other common sites include the small bones of the hands, feet, and spine [1]. It accounts for 10% to 12% of primary bone tumors [1]. It predominantly affects children and adolescents but can occasionally be seen in older individuals [1]. The tumor shows a male predominance with a male-to-female ratio of approximately 2:1 [1].

This osteoblastic lesion is typically characterized radiographically by a circular radiolucent nidus surrounded by a zone of reactive sclerosis [2]. Notably, six out of seven cases of OO occurring in the hand are initially misdiagnosed, likely due to their rarity in this location [3]. We report a case of OO arising in the distal phalanx of the ring finger in a 23-year-old male.

## Case presentation

**Figure 1:**
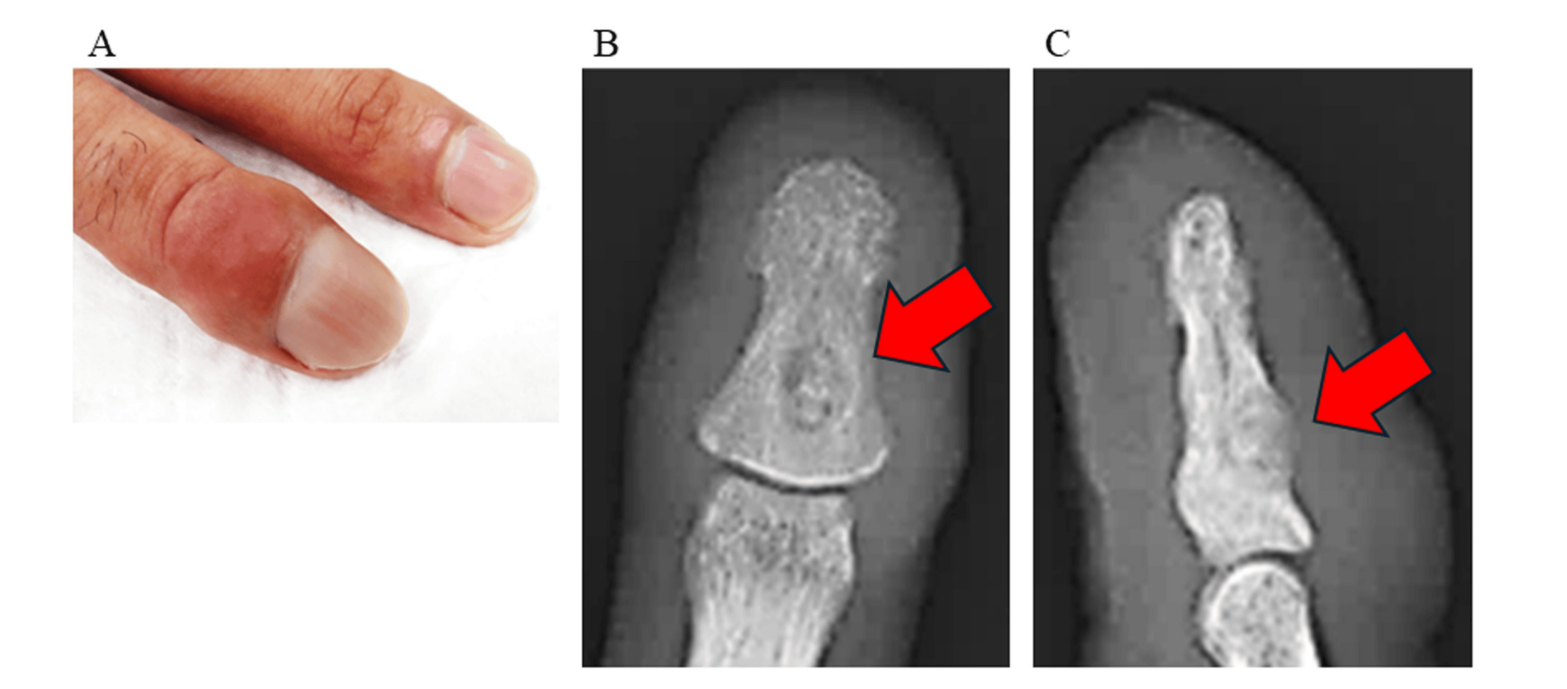
Gross appearance and preoperative radiographs A: Gross appearance of the distal right ring finger showing club-like thickening; B: Preoperative radiograph of anteroposterior view revealing a circular radiolucent lesion with internal calcification in the distal phalanx of the right ring finger (red arrow); C: Preoperative radiograph of lateral view demonstrating the same lesion on the dorsal aspect of the distal phalanx (red arrow)

The patient was a 23-year-old male who sustained a contusion to his right ring finger two months prior. He subsequently developed pain and swelling on the dorsal aspect of the distal phalanx of the same finger, which led him to seek medical evaluation at our hospital. Physical examination revealed diffuse tenderness over the dorsal distal phalanx, swelling, nail hypertrophy, and clubbing deformity (Figure 1, panel A). There was no restriction in the range of motion or sensory deficits. He took two 200 mg tablets of a cyclooxygenase-2 inhibitor twice daily and three 60 mg tablets of loxoprofen sodium three times daily for seven days each, but the effect was insufficient. The laboratory findings revealed no evidence of infection (Table 1). Radiographs demonstrated a radiolucent lesion with central calcification in the distal phalanx of the right ring finger (Figure 1, panels B and C).

**Table 1:** The laboratory findings The laboratory findings showed a C-reactive protein level of 0.015 mg/dL, a white blood cell count of 5,720/µL, and a neutrophil percentage of 49.4%, indicating no evidence of infection.

Parameter	Result	Reference range	Unit
C-reactive protein	0.015	<0.30	mg/dL
White blood cell count	5,720	3,500–9,000	/µL
Neutrophils	49.4	40–70	%

**Figure 2:**
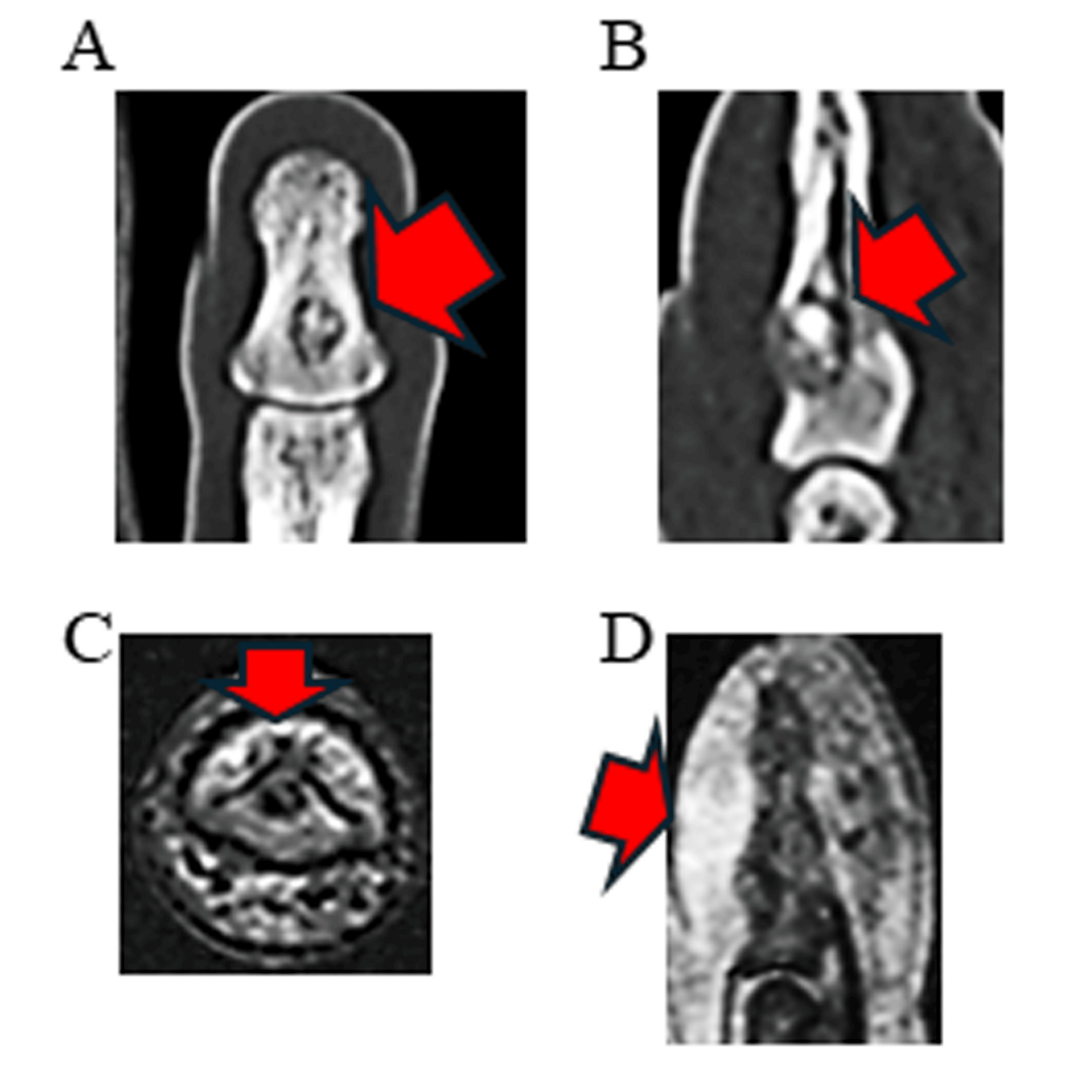
CT scans and MRI of the right ring finger A: Coronal CT image showing a circular bone resorption lesion with internal calcification within the distal phalanx (arrow); B: Sagittal CT image confirming the lesion on the dorsal aspect of the distal phalanx (arrow); C: Axial T1-weighted MRI revealing a low-intensity lesion in the distal phalanx (arrow); D: Sagittal T2-weighted MRI depicting the lesion and a high-intensity area adjacent to the dorsal side (arrow)

The CT confirmed this finding (Figure 2, panels A and B). The MRI revealed a low-intensity lesion on both T1- and T2-weighted images within the distal phalanx, with a high-intensity area adjacent to the dorsal side (Figure 2, panels C and D). 

**Figure 3:**
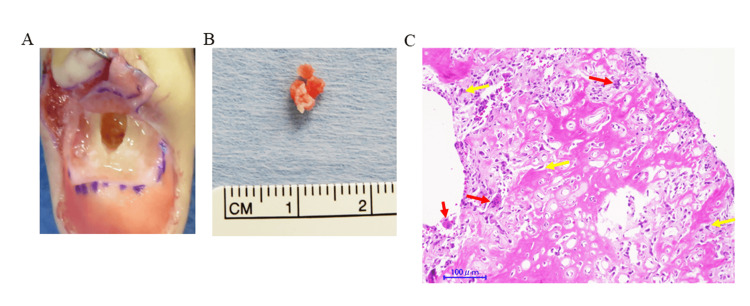
Intraoperative, specimen and histological findings A: Intraoperative view after removal of the nail plate to access the tumor site; the lesion was excised. B: Excised specimen showing a pale reddish-white lesion. C: Histopathological examination of the specimen revealed osteoid and bone formation with osteoblasts exhibiting mild nuclear irregularities (yellow arrows), as well as osteoclast-like multinucleated giant cells (red arrows).

Due to suspicion of a bone tumor, surgical resection was undertaken. The nail bed was preserved, and the tumor with thickened periosteum was excised (Figure 3, panel A). A bone cavity was observed dorsally in the distal phalanx, and internal soft tissues were removed (Figure 3, panel B). Histopathological examination of the specimen revealed osteoid and bone formation with osteoblasts exhibiting mild nuclear irregularities, as well as osteoclast-like multinucleated giant cells (Figure 3, panel C). The diagnosis of OO was confirmed.

**Figure 4:**
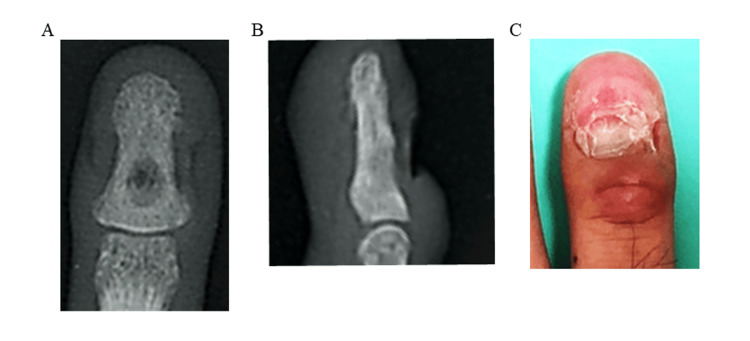
Postoperative radiographs and gross appearance of the distal right ring finger at two months A: Anteroposterior view showing no evidence of tumor recurrence and partial bone regeneration; B: Sagittal view confirming no recurrence and partial bone healing; C: Clinical photograph at two months post-surgery demonstrating resolution of the club-like finger thickening and nail regrowth

The patient's pain resolved immediately after surgery. Postoperatively, the distal interphalangeal (DIP) and proximal interphalangeal (PIP) joints were immobilized with a splint for two weeks. Two months after surgery, no recurrence was noted (Figure 4, panels A and B), and nail regrowth was observed (Figure 4, panel C).

## Discussion

In 1935, Jaffe first described the term "osteoid osteoma" for a small, round bone tumor characterized by a central core known as the nidus [4]. Elevated levels of prostaglandins within the nidus have been identified as key mediators of pain, particularly nocturnal pain that is typically relieved by nonsteroidal anti-inflammatory drugs (NSAIDs) [1,5]. Clinical symptoms often include localized tenderness, redness, and swelling, as observed in the present case [1,2,5].

In a retrospective study of 37 OO cases by Simon et al., 5.9% involved bones of the hand, with the phalanges being the most commonly affected site (59.5%), followed by metacarpal bones (24.3%) and carpal bones (16.2%) [6]. A literature review by Liu et al. reported that out of 289 cases of OO of the hand and wrist, only 19.4% involved the distal phalanx, with just eight cases specifically affecting the ring finger [6,7].

Osteoid osteomas are classified into three types based on their location: intramedullary (cancellous bone), cortical, and subperiosteal [8]. The cortical type is the most common, typically found in the shafts of long bones such as the femur and tibia. Intramedullary OOs, as seen in this case, are more frequently observed in the phalanges [2,5,9]. Delayed diagnosis and frequent misdiagnosis of OO are common, often leading to inappropriate initial treatments [3,10]. Burger et al. reported misdiagnoses including chondroma, benign schwannoma, monoarthritis, post-traumatic changes, osteochondroma, and infection [3].

Although the pathogenesis of OO remains unclear, 18% to 50% of patients with osteochondromas in the wrist and hand have a history of trauma. In addition, a recent review of 71 reported cases indicated that a history of trauma was observed in approximately 30% of the patients [11]. This history may contribute to misdiagnosis as a stress fracture or joint capsule injury, complicating the diagnostic process [3,10]. It should be noted, however, that the consideration of trauma history as an etiological factor is merely speculative. Also, the current case had no history suggestive of post-traumatic changes or osteomyelitis, necessitating differential diagnosis from other bone tumors.

Surgical treatment is considered for patients with severe pain or those unresponsive to NSAIDs. Available surgical options include radiofrequency ablation, CT-guided percutaneous excision, and en bloc resection [12]. En bloc resection or curettage remains the primary treatment modality, although curettage carries a higher risk of incomplete removal, which may increase recurrence risk and necessitate further surgery. Additionally, bone grafting is often required to prevent postoperative fractures [13]. While radiofrequency ablation is widely used for OO, its application in the hand is limited due to increased risks of thermal injury to neurovascular structures and the inability to obtain tissue for histopathological diagnosis [14]. Including recent reviews, previously reported cases of OO occurring in the digital bones of the hands and feet were reviewed with respect to location, age, treatment, and outcome (Table 2) [2-15].

**Table 2:** Summary of reported cases of OO in the phalanges of the hands and feet Cases were summarized regarding the site of occurrence, age, treatment, and outcome, showing a wide age distribution, frequent use of surgical excision, and generally favorable prognosis. OO: Osteoid osteoma, RFA: Radiofrequency ablation

Author & year	Site	Age	Treatment	Outcome
Tsang & Wu (2008) [2]	Phalangeal bone	Mainly 20s–30s	Complete surgical excision	Favorable; recurrence rare
Burger & McCarthy (2004) [3]	Phalanges	Mostly young patients	Difficult diagnosis; managed surgically	Favorable; remission with proper treatment
Jaffe (1935) [4]	Multiple sites	Wide range	Surgical excision	Generally favorable; rare recurrence
Daher, et al. (2021) [5]	Distal phalanx	Exemplary case	Surgical treatment	No recurrence; symptom improvement
Simon, et al. (2014) [6]	Phalanges	Middle-aged to elderly patients	Various approaches, including surgery and diagnostic assessment	Majority showed successful outcomes
Liu, et al. (2017) [7]	Proximal phalanx	Late teens–30s	Surgical excision	Good; preserved function
Liu, et al. (2006) [8]	Juxta-articular region (complex site)	Middle-aged	Individualized, mainly surgical	Occasionally complex; requires follow-up
Ambulgekar & Ghag (2022) [9]	Distal phalanx of the little finger	20s–40s	Surgical excision	Favorable outcome
Hashemi, et al. (2011) [10]	Phalanges	Not specified	Surgical excision (mainly for diagnostic purpose)	–
Noordin, et al. (2018) [11]	Various bones	Wide range	Non-surgical management, including radiofrequency ablation (RFA)	High success rate; low recurrence
Becce, et al. (2011) [12]	Distal phalanx of the middle finger	About 30s	Surgical excision	Symptom resolution
Rosenthal, et al. (1998) [13]	Various bones	Wide range	Comparison of RFA and surgical excision	High success with RFA; minimally invasive
Alruwaii, et al. (2023) [14]	Phalanges and foot bones	7–64 (median 23)	Mainly surgical excision; RFA when indicated	Mostly favorable; majority healed with low recurrence
Meyer, et al. (2023) [15]	phalanges / digital bones		Surgical excision/ RFA	Mostly favorable; majority healed with low recurrence/
The current case	Distal phalanx of the ring finger	23	Surgical excision	No recurrence

The cases demonstrated a wide age distribution, a predominance of surgical excision as the treatment method, and generally favorable prognoses, including the present case. In this case, curettage was performed to establish a definitive diagnosis. Careful postoperative monitoring is essential to detect any recurrence. A longer follow-up period of six to 12 months is required to confirm recurrence more definitively.

## Conclusions

Osteoid osteoma in the distal phalanx is exceptionally rare, and its diagnosis can be challenging due to its atypical presentation and frequent misdiagnosis as other fingertip lesions. This case highlights the importance of maintaining a high index of suspicion for OO when evaluating persistent pain and swelling in the phalanges, especially following trauma. Accurate identification through imaging and histopathology enables appropriate surgical intervention, as demonstrated by the excellent postoperative outcomes in this report. Complete excision of the nidus led to immediate relief of symptoms and prevented functional compromise or tumor recurrence at follow-up. Incorporating OO into the differential diagnosis of distal phalanx lesions may help clinicians avoid delayed treatment and achieve optimal patient recovery.
